# Pilot phase III immunotherapy study in early-stage breast cancer patients using oxidized mannan-MUC1 [ISRCTN71711835]

**DOI:** 10.1186/bcr1505

**Published:** 2006-06-15

**Authors:** Vasso Apostolopoulos, Geoffrey A Pietersz, Anastasios Tsibanis, Annivas Tsikkinis, Heleni Drakaki, Bruce E Loveland, Sara J Piddlesden, Magdalena Plebanski, Dodie S Pouniotis, Michael N Alexis, Ian F McKenzie, Stamatis Vassilaros

**Affiliations:** 1 Immunology and Vaccine Laboratory, Burnet Institute at Austin, Heidelberg, Victoria, Australia; 2 Prolipsis Medical Center, Athens, Greece; 3 Institute of Biological Research and Biotechnology, The National Hellenic Research Foundation, Athens, Greece

## Abstract

**Introduction:**

Mucin 1 (MUC1) is a high molecular weight glycoprotein overexpressed on adenocarcinoma cells and is a target for immunotherapy protocols. To date, clinical trials against MUC1 have included advanced cancer patients. Herein, we report a trial using early stage breast cancer patients and injection of oxidized mannan-MUC1.

**Method:**

In a randomized, double-blind study, 31 patients with stage II breast cancer and with no evidence of disease received subcutaneous injections of either placebo or oxidized mannan-MUC1, to immunize against MUC1 and prevent cancer reoccurrence/metastases. Twenty-eight patients received the full course of injections of either oxidized mannan-MUC1 or placebo. Survival and immunological assays were assessed.

**Results:**

After more than 5.5 years had elapsed since the last patient began treatment (8.5 years from the start of treatment of the first patient), the recurrence rate in patients receiving the placebo was 27% (4/15; the expected rate of recurrence in stage II breast cancer); those receiving immunotherapy had no recurrences (0/16), and this finding was statistically significant (*P *= 0.0292). Of the patients receiving oxidized mannan-MUC1, nine out of 13 had measurable antibodies to MUC1 and four out of 10 had MUC1-specific T cell responses; none of the placebo-treated patients exhibited an immune response to MUC1.

**Conclusion:**

The results suggest that, in early breast cancer, MUC1 immunotherapy is beneficial, and that a larger phase III study should be undertaken.

## Introduction

Outcomes of breast cancer treatment have improved, mostly as a result of earlier diagnosis. Although newer modes of therapy are being applied, traditional therapy involving surgery, radiotherapy and chemotherapy, followed by long-term antioestrogen therapy continues to be used. Immunotherapy could be a useful adjunct to conventional therapy, particularly in an adjuvant setting and (as shown here) in patients with early disease and no metastasis. New therapeutic procedures in breast cancer are usually tried in patients with advanced disease, who may be appropriate candidates for cytotoxic drugs. However, such patients may be unable to respond appropriately to immunotherapy, given that an intact and competent immune system is required to induce a therapeutic immune response.

Treatment of cancer with immunotherapy has been the goal of many researchers since the advent of effective immunization against infectious diseases. Previously, tumour antigens were not easily identified, but currently identified antigens include glycoproteins and glycolipids (for example, gangliosides), developmental and over-expressed antigens (for example, CEA (carcinoembryonic antigen), gp75, MAGE, tyrosinase, melan-A, mucin [MUC]1), and mutated oncogenes (for example, p53, HER-2/neu, ras) [1]. Our laboratory has focused on MUC1 as a target for tumour immunotherapy. Mucins (such as MUC1) are high-molecular-weight glycoproteins that are secreted by many epithelial cells such as breast, ovary, colon and pancreatic carcinomas. MUC1 is of interest and a potential target for tumour immunotherapy for the following reasons: there is an up to 100-fold increase in the amount of mucin present on cancer cells compared with normal cells; MUC1 has a ubiquitous rather than focal cellular distribution; and MUC1 has altered glycosylation, revealing peptide epitopes not easily identified in normal mucins.

Cloning of the cDNA for MUC1 and definition of the structure revealed that the molecule is transmembranous, with a relatively large extracellular domain and a cytoplasmic tail. It was discovered that most of the immunogenicity (in terms of antibody production) resided in a repeated (variable number of tandem repeats [VNTR]) 20-amino-acid peptide (PDTRPAPGSTAPPAHGVTSA) domain in the extracellular portion of the molecule [2-4]. Such studies of immunogenicity in mice would not be relevant to humans other than the findings that, in humans, MUC1 can stimulate T cells in breast, pancreatic and ovarian cancers [5-7]. Restricted cytotoxic T lymphocytes in humans with breast cancer and in pregnancy, as well as in mice, have also been detected [8-10]. In addition, T cell immunity and B cell immune responses to selected epitopes of MUC1 from ovarian, breast, pancreatic and colon cancer patients have been demonstrated [11-13], as have circulating immune complexes to MUC1 in the serum of breast and ovarian carcinoma patients [14]. In mice, we demonstrated that a 20-mer MUC1 VNTR (made as a fusion protein comprising five VNTR repeats), when coupled to oxidized mannan (that is, oxidized mannan-MUC1 fusion protein [M-FP]), generates H2 restricted cytotoxic T lymphocytes, which protect mice against challenge with MUC1^+ ^mouse tumours [9,15-22].

Over the past 12 years, we have injected many patients with different MUC1 formulations in an effort to induce protective and therapeutic immunity, aiming to reproduce the same effect as seen in mice [1,9,16-20,23-35]. In more than 250 patients with advanced cancer, moderate cellular immune responses and substantial antibody responses were noted [36-39]. However, clinical responses were not apparent in these patients, possibly because of the advanced and immunocompromised state of their disease. Therefore, in the present study we aimed to evaluate the effect of M-FP in patients with early disease. Specifically, these patients had stage II disease with fewer than four lymph nodes involved, all of which had been removed, and had no evidence of disease and were entirely healthy at the start of the trial. Although a small number of patients were recruited into this randomized, double-blind pilot study, the early results reported here are promising and justify the performance of a larger study.

## Materials and methods

### Patient recruitment

A total of 31 postmenopausal women with stage II breast cancer (oestrogen receptor positive and with involvement of no more than four ipsilateral nodes) and no evidence of distant disease participated in the study. All patients received tamoxifen. The patients were selected and treated at Prolipsis Medical Center, Athens, Greece. The study was initiated once ethics approval had been granted by the National Drug Administration, Greece (EOF) (26 September 1997, no. 27581). The inclusion and exclusion criteria are summarized in Table 1.

Staging of the disease was done by conventional measurements, chest radiography, bone scan, and ultrasonography of upper and lower abdomen (to rule out distant metastases). The age of the patients was 51–81 years (mean 59.5 years). The histologic type of the tumor in 28 patients was infiltrating adenocarcinoma. The grading was as follows: grade I in six cases, grade II in 19 cases, and grade III in three cases. In three patients the histologic type was infiltrating lobular carcinoma with no grading. All tumours were positive for oestrogen receptor. The size of the primary tumour ranged from 0.5 to 3.2 cm (mean 1.8 cm). The total number of excised lymph nodes per patient ranged from eight to 29 (mean 18.3). The positive nodes per patient ranged from one to four (mean 1.7) (Table 1).

### Preparation of MUC1 fusion protein, pVNTR and conjugation to oxidized mannan

The MUC1 fusion protein (FP), consisting of glutathione-*S*-transferase (GST; 26 kDa) and three MUC1 VNTR repeats (PDTRPAPGSTAPPAHGVTSA) plus two flanking homologous sequences (12 kDa), was induced in *Escherichia coli *and purified (as described previously) [40]. Mannan (Sigma, St. Louis, MO, USA) was oxidized and conjugated to FP to form M-FP (oxidized mannan-FP; as described previously) [19,20,36,38]. An alternative source of the antigen, not containing GST, was prepared to assay VNTR-specific immune responses in ELISpot and ELISA assays. pVNTR recombinant protein was expressed in *E. coli *using the pTrcHisB vector (Invitrogen, Carlsbad, CA, USA) containing the pDF9.3 insert and purified on a Nickel-NTA column (Qiagen, Melbourne, VIC, Australia). pVNTR contains the same MUC1 sequence as FP (above) but with no GST and His_6 _for purification. M-FP and pVNTR were tested for sterility (presence of bacteria and fungi; Austin Health, Department of Medical Microbiology, Heidelberg, VIC, Australia) and bacterial endotoxin activity (EML Group of Laboratories, Melbourne, VIC, Australia). The MUC1 was conjugated to oxidized mannan (to target the mannose receptor) [16,24] using periodate; this leads to the formation of aldehydes, which are critical for rapid exit from endosomes and entry into the major histocompatibility complex class I pathway [18]. Placebo consisted of the conjugation buffer (bicarbonate buffer; pH 9.0) alone. The vaccine was prepared under Good Laboratory Practice (GLP) guidelines at the Burnet Institute (Austin, Melbourne, Australia), and endotoxin levels were above 0.06 and below 0.6 EU/ml. M-FP vaccine was also tested for activity using ELISA binding assays and by sodium dodecyl sulphate gels. The vials were number coded by randomization and sent to Prolipsis Medical Center (Athens, Greece) from the Burnet Institute, and stored at -20°C; the codes were held by Burnet Institute.

### Immunotherapy schedule, blood samples and assessment of clinical status

Commencing 3 months after surgery, 31 patients with early-stage breast cancer were given seven injections at 2-week intervals of M-FP or placebo for the first 12 weeks and then at 6 and 9 months (Table 2). Blood samples were taken before and at intervals after M-FP or placebo injections. Peripheral blood mononuclear cells (PBMCs) were isolated from heparinized blood by centrifugation with Ficoll-Paque and stored in liquid nitrogen at the National Hellenic Foundation until they were sent in dry ice to the Burnet Institute for immunological analysis (antivaccine IFN-γ T cell immunity). Serum samples were also collected from patients and stored at -20°C to monitor antibody responses (Table 2). Follow-up tests included laboratory tests at Prolipsis Medical Center, chest radiographs, and upper and lower abdominal ultrasound every three months for five years. Bone scans and mammography were performed every year for up to 7 years/10 months (Table 2). Patients' breast tumours were not assessed for MUC1 expression in this trial; however, we have found that more than 90% of breast cancers express MUC1, and so it is likely that most of the patients were positive for MUC1 expression [3,4]. We have tested for MUC1 expression in dendritic cell/M-FP injected patients [41], and in the trial currently in progress.

### Immunological assays

#### ELISpot cytokine assays (measurement of IFN-γ secretion by T cells)

Plates (96-well, MAIPS S45; Millipore, Sydney, Australia) were precoated with 50 μl anti-human IFN-γ antibody (1D1K; Mabtech, Meblbourne, Australia). Triplicate wells were set up for each condition. Each test well contained 0.5 × 10^6 ^viable thawed PBMCs in 100 μl RPMI-1640 medium supplemented with glutamine, penicillin, streptomycin and 2.5% (vol/vol) human AB serum (Valley Biomedical, Winchester, VA, USA), plus 25 μl control additive or antigen (purified protein derivative (PPD; Tuberculin, Staten Serum Institute, Copenhagen, Denmark; positive control antigen), recombinant pVNTR (specific test antigen), or no antigen (negative control]) to a final concentration of 20 μg/ml. The plates were incubated for 18 hours at 37°C in 5% carbon dioxide. The cells were removed with sequential washes of phosphate-buffered saline (PBS) containing 0.1% vol/vol Tween 20 then PBS, and the plates were incubated with biotin-conjugated anti-human IFN-γ antibody (7-B6-1; Mabtech) for 2 hours at room temperature before washing. Bound cytokine was visualized using 1 μg/ml streptavidin-alkaline phosphatase conjugate (Mabtech) incubated for 2 hours at room temperature, and then incubation with the colorimetric AP detection kit in accordance with the manufacturer's instructions (BioRad, Salt Lake City, UT, USA). Cytokine spots were counted using the AID ELISpot Reader system (Autoimmun Diagnostika GmbH, Strassberg, Germany). PPD was used as a positive control antigen that all patients were expected to respond to, particularly to validate PBMC sample viability and reactivity.

#### ELISA assays (measurement of IgM and IgG antibody levels)

Anti-MUC1 VNTR antibodies were measured using ELISA against pVNTR recombinant protein. ELISA plates (Costar, Temecula, CA, USA) were coated with 50 μl of a 5 μg/ml antigen solution in 0.05 mol/l sodium carbonate buffer (pH 9.6) for 2 hours at 37°C. Plates were washed five times in 0.1% vol/vol Tween 20 in PBS and then in PBS, and were blocked with 2.5% vol/vol human AB serum in PBS (100 μl) for 1 hour at 37°C. The plates were washed and test sera, diluted 1/40 in 1% vol/vol human AB serum-PBS, were added and incubated for 2 hours at 37°C. Following five washes with 0.1% (vol/vol) Tween 20 in PBS and five washes with PBS, bound serum antibodies were visualized with 50 μl of isotype and class-specific sheep anti-human immunoglobulin, anti-IgG, or anti-IgM antisera, each labelled with horseradish peroxidase (Silenus Laboratories, Hawthorn, Australia) and diluted to 1:500 in 1% (vol/vol) AB serum and incubated for 1 hour at 37°C. The plates were washed and developed with 0.03% vol/vol 2,2-azino-di-(3-ethylbenzthiozoline sulfonate) in 0.1 mol/l citrate buffer (pH 4.0). Absorbance was measured at 405 nm using an ELISA plate reader (FLUOstar Optima plate reader; BMG Labtechnologies GmbH, Offenberg, Germany). A positive control serum was selected from a panel, being from a patient previously immunized with M-FP (IFCM10_#2EC) who had developed an anti-VNTR IgM and IgG antibody response. The negative control serum was selected from a panel, being from a normal individual (ARI_#S2P) with no detectable anti-VNTR antibody. The antibody reactions are presented as percentage positive of test sera compared with positive control sera, after validating that there was no nonspecific antibody binding of test or control sera (diluted 1/40) to empty wells and the negative control reaction was under 15% compared with the positive serum.

### Statistical analysis of protective efficacy against relapse

The data for the placebo and M-FP group was plotted as Kaplan-Meier survival curves using the PRISM program [42].

## Results

### Clinical findings and patient characteristics

The first patient was injected on 13 December 1997 and the last patient on 17 July 2000. On 18 June 2003 the vaccine code was broken, identifying the 16 patients who had been injected with M-FP (Table 3) and the 15 who had been injected with placebo (Table 4). Of the M-FP injected patients none of 16 had relapse of disease and four out of 15 placebo patients had recurrent disease. Three out of 31 patients did not complete the injection programme, all of whom were in the placebo group; one patient refused to continue in the programme after the eighth injection and developed local metastases 2 months later (Table 4). The second patient stopped the treatment after three injections and the third patient after the first injection. The follow-up period ranged from 60 to 99 months. As of May 2006 there have been no changes to the clinical findings reported here.

The characteristics of the patients in the M-FP vaccinated group, and their pathology and follow up are presented in Table 3. As noted in Table 3, the age of the patients ranged from 52 to 78 years (mean 59.9 years). Modified radical mastectomy was performed on six patients and partial mastectomy with radiation on 10 patients. The histological type of the tumour was infiltrating adenocarcinoma. In three cases the histological type was infiltrating lobular carcinoma. The grading was I in four patients, II in eight and III in one. In the three cases of lobular carcinoma we had no grading data. Tumour size ranged from 0.8 to 3 cm (mean 1.5 cm). The total number of excised lymph nodes per patient ranged from eight to 29 (mean 17.9). The number of positive nodes per patient ranged from one to four (mean 1.8). Mild skin redness was noticed in six patients at the area of vaccination and needed no treatment. The follow up of these patients ranged from 60 to 90 months (mean 75.6 months) and no signs of recurrence were evident.

The characteristics of the patients in the placebo group, and their pathology and follow up are presented in Table 4. The age of the patients ranged from 51 to 81 years (mean 68.8 years). Modified radical mastectomy was performed in six patients and partial mastectomy, axillary dissection and radiation in nine patients. Tumour size ranged from 1.5 to 3.2 cm (mean 2.1 cm). The histological type of the tumor in all 15 patients was infiltrating adenocarcinoma. The grading was I in one patient, II in 12 patients and III in two patients. The total number of dissected lymph nodes per patient was 11 to 27 (mean 18.9). The number of positive nodes per patient ranged from one to three (mean 1.6). Mild skin redness was noticed in four patients and needed no treatment. Four patients from this group presented with metastatic disease. One patient developed liver metastases 9 months after surgery and died 1.5 years later. A second patient developed bone metastases 24 months after surgery and died 3 years later. Two other patients developed bone and local metastases 18 and 19 months after surgery and they are still living with disease 76 and 90 months after surgery. The remaining 11 patients, with follow up ranging from 75 to 99 months (mean 91.0 months), are living free from disease. Inspection of characteristics between the two groups indicates that they are appropriate for statistical comparison.

### ELISpot assays of IFN-γ secretion by T cells

**Figure 1:**
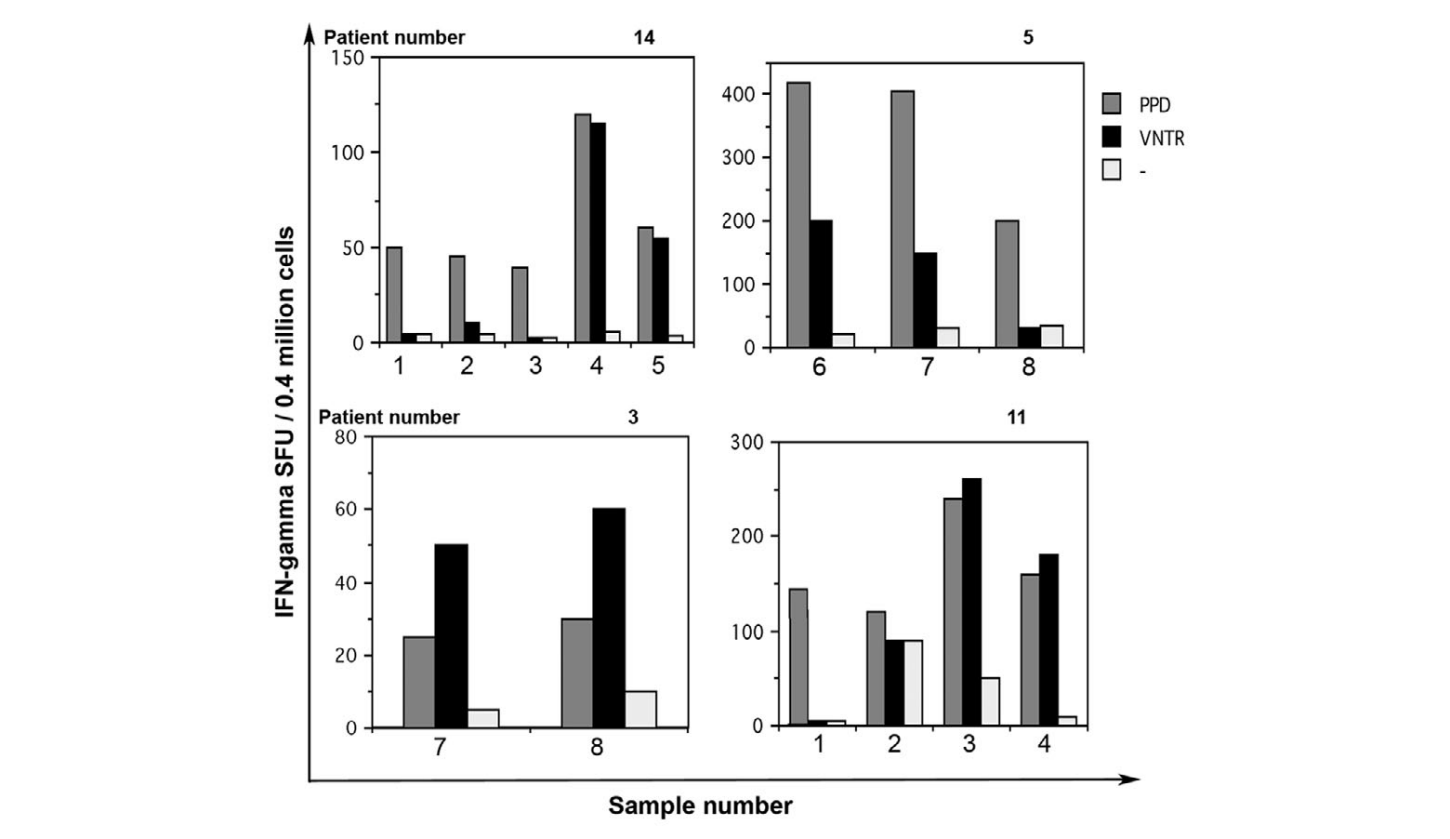
IFN-γ secreting T cells in patients immunized with M-FP. Patient numbers 14, 5, 3 and 11 are shown. Responses to VNTR (pVNTR (which contains five VNTR repeats with no GST); black), PPD (internal positive control; grey) and no antigen (negative control; white). 1 = first injection; 2 = week 6 (2 weeks after the third injection); 3 = week 12 (2 weeks after the sixth injection); 4 = 6 months (3 months after the seventh injection); 5 = 9 months (3 months after the eighth injection); 6 = 1 year (3 months after the final (ninth) injection); 7 = 2 years, and 1 year and 3 months after the final injection; 8 = 3 years, and 2 years and 3 months after the final injection. GST, glutathione-*S*-transferase; IFN, interferon; M-FP, oxidized mannan conjugated to MUC1 fusion protein; PPD, purified protein derivative; SFU, spot forming units; VNTR, variable number of tandem repeats (from MUC1 sequence).

Because of cell storage problems and transport of frozen cells from Greece to Melbourne, only 63% (10/16) and 53% (8/15) of samples in the vaccinated and placebo groups exhibited viability in excess of 80% upon recovery and were tested by ELISpot. The cells that were viable were functionally active because they responded to PPD (Figure 1) and all these samples were validated by proliferative response to Phytohemagglutinin (data not shown) before they were evaluated using the assays. IFN-γ secreting T cells were generated in 40% (4/10) of samples from patients immunized with M-FP (patient numbers 3, 5, 11 and 14; Table 3). T cells were shown to be specific for MUC1 VNTR (pVNTR). Examples of responses in patients (3, 5, 11 and 14) receiving M-FP are shown in Figure 1. Patient 14 exhibited no IFN-γ T cell responses to pVNTR in samples 1, 2 and 3 but significant responses in samples 4 and 5 (sample numbers shown in Table 2; Figure 1). Patient 5 had high IFN-γ T cell responses to pVNTR in samples 6, 7 and 8 but, because of to low cell viability, we were unable to test samples 1–5 (Figure 1). Likewise, for patient number 3, only samples 7 and 8 are shown. Sample 6 corresponds to 1 year after the first injection, and samples 7 and 8 correspond to blood taken after 2 and 3 years, respectively. Thus, in patient 5 T cell responses were detectable up to 2 years after the first injection, and in patient 3 they were detectable 3 years after the first injection. We tested pVNTR responses in more 20 nonimmunized individuals and all exhibited no responses to pVNTR (data not shown). Thus, it is probable that the responses to pVNTR would be negative in sample 1 (taken on the day of vaccine injection) from patients 3 and 5. Patient number 11 had detectable pVNTR IFN-γ responses in samples 2, 3 and 4. Of the eight samples tested from the placebo group, no T cells reactive with MUC1 (pVNTR) were detectable (data not shown). Furthermore, we did not detect IFN-γ responses by ELISpot among patients receiving placebo or in pretreatment samples (for example, sample 1; Figure 1). The ELISpot results are statistically significant (*P *< 0.05) for the comparison with sample number 1 in patients 11 and 14, and for the comparison with negative control in all patients.

### Measurement of antibodies by ELISA assays

**Figure 2:**
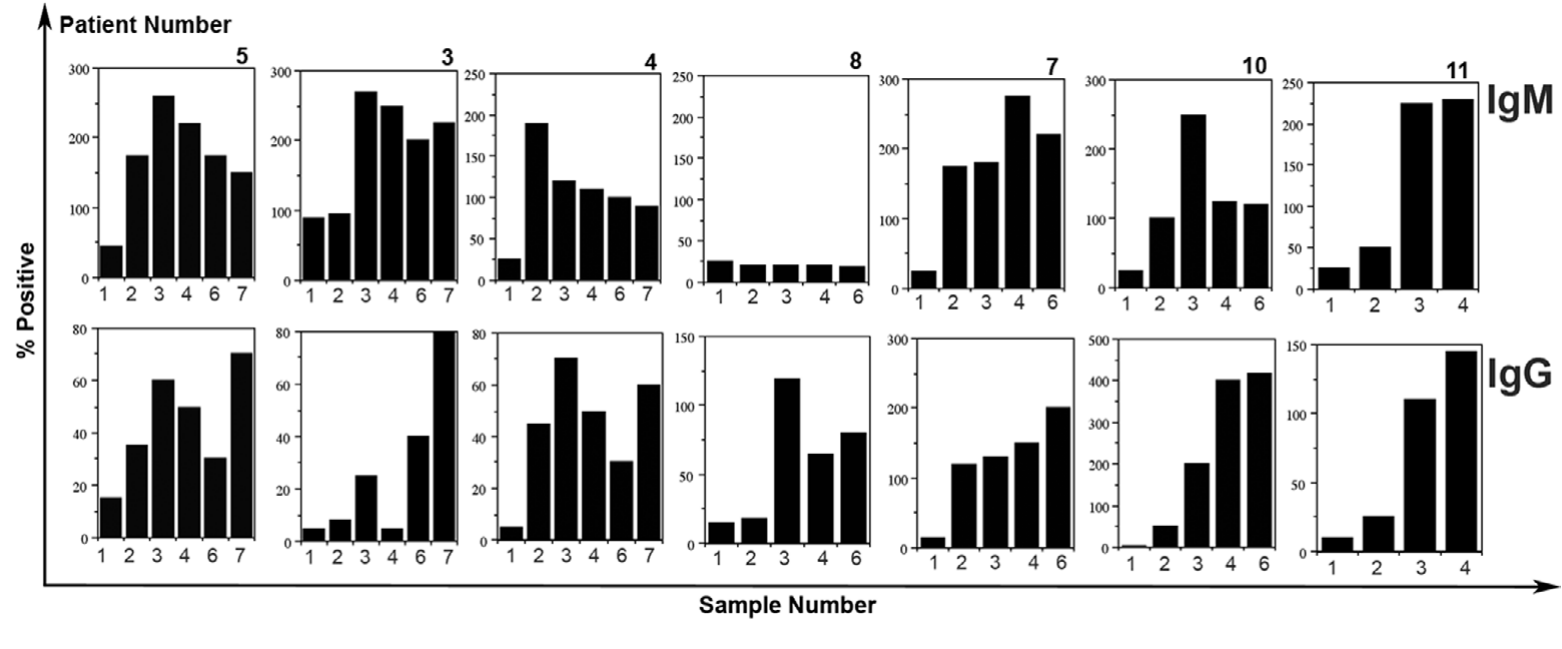
Antibody (IgM and IgG) responses to VNTR in patients immunized with M-FP. Representative examples from patients 3, 4, 5, 7, 8, 10 and 11. 1 = first injection; 2 = week 6 (2 weeks after the third injection); 3 = week 12 (2 weeks after the sixth injection); 4 = 6 months (3 months after the seventh injection); 6 = 1 year and 3 months after the final (ninth) injection; 7 = 2 years, and 1 year and 3 months after the final injection. A positive control serum was selected from a panel, being from a patient previously immunized with M-FP (IFCM10_#2EC) who had developed an anti-VNTR antibody response. Negative control serum was selected from a panel, being from a normal individual (ARI_#S2P) with no detectable anti-VNTR antibody. The antibody reactions are presented as percentage positive of test sera compared with positive control sera (where the negative control serum reaction was <15% of positive serum). M-FP, oxidized mannan conjugated to MUC1 fusion protein; VNTR, variable number of tandem repeats (from MUC1 sequence).

Anti-VNTR serum antibodies were induced in nine out of 13 participants who had received M-FP. These were initially IgM, and seroconverted to IgG and persisted up to 12–24 months after immunization. Representative antibody reactions from seven patients (patients 3, 4, 5, 7, 8, 10 and 11) are shown in Figure 2. Sample 1 denotes the pretreatment sample from each patient. Thus, antibodies were detected only after vaccination. No detectable antibody levels were found in the patients receiving placebo in all samples tested (not shown). We did not detect antibodies to MUC1 in the placebo group or in the pretreatment samples of M-FP immunized patients (sample 1; Figure 2). However, we note that background IgM levels in sample 1 were higher in patient 3, which increased threefold after injections with M-FP compared with all other patients' samples. The antibody results are statistically significant (*P *< 0.05) for the comparison with sample 1 in each patient group.

### Statistical analysis of protective efficacy against relapse

**Figure 3:**
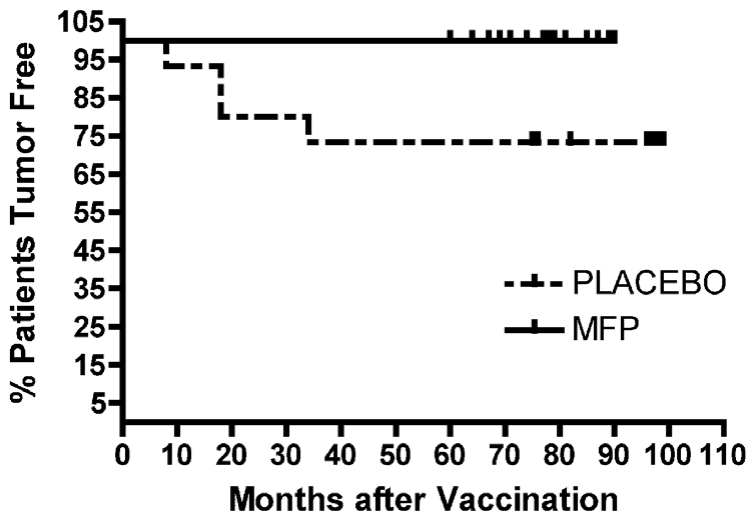
Kaplan-Meier survival curves. The PRISM program was used to construct the curves for the placebo and M-FP immunized patients. M-FP, oxidized mannan conjugated to MUC1 fusion protein.

The data for the placebo and M-FP group were plotted as Kaplan-Meier survival curves using the PRISM program [42]. All patients enrolled in the trial were assessed and included the patients who did not receive the complete treatment. All of the patients with relapses and no relapses were analysed, with time of observation ranging from 60 to 99 months. The vertical bars are the last day of follow up. The placebo and M-FP curves were compared using the log rank test and found to differ significantly (*P *= 0.0292 and χ^2 ^4.812; Figure 3).

## Discussion

In this long-term, double-blind study of 31 individuals with stage II breast cancer and no evidence of disease (16 injected with M-FP and 15 with placebo), patients receiving M-FP vaccination appeared to benefit in terms of protection against relapse; none of 16 had a recurrence, but four out of 15 placebo patients had recurrent disease after 7 years and 10 months (December 1997 to October 2005). Although the number of patients in this study is small, the results are statistically significant (*P *= 0.0292). Most of the treated patients exhibited immunity to MUC1 VNTR, whereas none of the placebo patients had such immune responses. Thus, M-FP appears to confer the survival/disease-free interval advantage in patients with early breast cancer. Importantly, and in contrast to other studies, the findings presented here for vaccination of patients with early disease provide justification for performance of a larger study. Indeed, a strategy of using a small number of patients to obtain an indication of response would be worth considering for immunotherapy trials, in which virtually all patients should exhibit an immune response if the therapy is working, and a high proportion of those should exhibit an anti-tumor response; this contrasts with cancer chemotherapy, in which fewer patients are likely to respond.

The study is of interest for several reasons. First, we highlight the characteristics of the patients evaluated in the present study; these patients had early disease, they presented with a primary lesion, they had lymph nodes surgically removed, and at the time of commencement of the trial they had no evidence of disease. We consider these characteristics to be crucial if immunotherapeutic studies are to yield useful findings. In another trial [43], a potentially therapeutic monoclonal antibody (CO17-1A) was used in 83 patients with minimal residual colorectal cancer (Dukes C) after surgery, and some patients remained tumour free for many years. Furthermore, in a placebo-controlled clinical trial using NY-ESO-1 protein (cancer testis protein) with ISCOMATRIX adjuvant in early-stage melanoma patients [44], immunized patients exhibiting antibody, delayed-type hypersensitivity, and CD4^+ ^and CD8^+ ^T cell responses appeared to have superior clinical outcomes to those treated with placebo or protein alone. Unfortunately, most cancer trials involve patients with advanced disease, selected on the basis of traditional therapy involving toxic chemotherapeutic agents. However, such patients are likely to have poor immune responses, significant tumour bulk, limited time for the induction and effector phases of specific immunity, and are the least likely to have therapeutic benefit from immunotherapy regimens. We do not advocate abandonment of the traditional approach of phase I studies, in which the primary end-point is assessment of toxicity, but we do recommend that these pilot studies of immunotherapeutic agents be quickly followed by larger studies in patients with early disease. In our experience to date, more than 250 other patients have received M-FP by direct injection but all had advanced disease; no toxic effects were detected but – importantly – none had objective clinical responses [36-39]. We hope that the present study will encourage use of immunotherapy in patients with early disease, but we note the long follow-up time required (and the large number of patients) if meaningful results are to be to obtained.

A second reason why this study is of importance is that some of the patients had a cellular immune response (of the samples that could be tested), and most had a significant antibody response. In contrast to patients from other clinical trials of M-FP [36-39,41], we did not detect IFN-γ, cytotoxic T lymphocytes, or proliferation of T cells to pVNTR in pretreatment samples. Interestingly, pre-existing peptide-reactive T cell responses (IFN-γ) to MUC1, as measured using quantitative polymerase chain reaction, have been detected in normal donors and in patients with primary breast cancers [45]. However, the responses were against different MUC1 antigens, namely short peptide epitopes MUC1_950–958 _(STAPPVHNV) and MUC1_12–20 _(LLLLTVLTV), which are outside the MUC1 VNTR region and were not incorporated in our M-FP vaccine. We did not detect anti-MUC1 antibody levels in pretreatment samples in other M-FP clinical trials either [36-39,41]. Although our finding is contradictory to those of previous studies [11,12,46], this is possibly due to differences in ELISA methodology or the antigen used to coat the ELISA plates (recombinant pVNTR in our studies versus peptide in other studies), which would mean that the conformation of the antigen is different [11]. We also noted that no immunized patients exhibited strong antibody responses after the limited course of subcutaneous injections (that is, titres were <1/40 to 1/80 only), and so the results shown for 1/40 serum dilutions are specific and represent essentially maximum reactivity.

It is difficult to conclude firmly that antibody and/or T cell responses led to nonrecurrence of tumour, but it is possible that the persistent presence of antibody would bind to small metastatic deposits, leading to their eradication, whereas with large deposits there is the difficulty of penetration of the antibody into the tumour cell mass; similar comments apply to T cells. We note a study [47] in which it was demonstrated that the occurrence of MUC1 antibodies without immunization in early breast cancer patients (stages I and II) were associated with significant benefit in terms of disease-specific survival. Patients immunized in our trial and who had developed IgM and IgG antibodies appeared to have benefit, with significantly delayed recurrence. We await the results of a larger study (in excess of 360 patients, which is in progress (EOF ethics approval, 30 December 2004; no. 64055)) before we draw firm conclusions on the role of anti-MUC1 immunity and lack of recurrence.

Finally, although antibody production and cellular immunity occurred only in the immunized group, there was no control group with oxidized mannan only; it is theoretically possible that the mannan, by cross-linking mannose receptors on macrophages and dendritic cells, could lead to their activation. An endogenous immune response could eradicate tumour deposits or activate macrophages, which could eradicate tumour cells because macrophages are known to be cytocidal to tumour cells in the absence of any immune response. However, in many experimental studies mannan mixed with MUC1 FP failed to induce the necessary immune responses and tumour protection to have any impact on tumour cell growth in mice [19,20]. Therefore, this mechanism is unlikely to account for the results presented here.

## Conclusion

This study provides important justification for studies of immunotherapeutic studies in patients with early/minimal/no evidence of disease. It also suggests that M-FP confers some benefit. A larger trial is now in progress (multicentre trial to include >360 patients), which will yield further data.

## Abbreviations

ELISA = enzyme-linked immunosorbent assay; FP = MUC1 fusion protein; GST = glutathione-*S*-transferase; IFN = interferon; MUC = mucin; M-FP = oxidized mannan conjugated to MUC1 fusion protein; PBMC = peripheral blood mononuclear cell; PBS = phosphate-buffered saline; PPD = purified protein derivative; pVNTR = five VNTR repeats with no GST; VNTR = variable number of tandem repeats (from MUC1 sequence).

## Competing interests

The authors declare that they have no competing interests.

## Authors' contributions

VA, GAP, IFM and SV participated in the design of the study. VA and GAP supervised production of Good Lab Practice (GLP) grade vaccine/placebo. SV collected blood samples and MNA isolated and stored sera and PBMCs. VA, GAP, BEL, SJP, MP and DSP all assessed and performed immunological analysis of blood samples. SV performed the clinical trial at Prolipsis Medical Center, and HD, A Tsibanis and A Tsikkinis were involved in the clinical trial management, patient recruitment, injection and planning. VA and GAP also performed the statistical analysis. All authors read and approved the final manuscript.

## Figures and Tables

**Table 1:** Inclusion and exclusion criteria for participation in the study

Criteria	Details
Inclusion	Postmenopausal women (no menstrual period for >12 months)
	Histological proven adenocarcinoma of the breast treated primarily by modified radical or partial mastectomy and axillary dissection followed by radiotherapy of the residual breast
	No more than four ipsilateral lymph nodes with metastases, not extending into the surrounding tissue and surgical margin free from disease
	Tumor tissue positive for oestrogen receptor
	Tamoxifen 20 mg/day commencing within 3 months of breast surgery and to continue for 5 years (provided by A. Dervos – G Dimitrakopoulos &Co OE)
	Adequate bone marrow function (white blood cell count >4.0 × 10^9^/l, haemogoblin >100 g/l, platelet count >100 × 10^9^/l)
	Adequate liver function (billirubin <60 μmol/l [that is, under three times the upper limit of normal)
	Adequate renal function (creatinine <140 μmol/l)
	Life expectancy >12 weeks
	ECOG (Eastern Cooperative Oncology Group) status between 0 and 2 (In bed <50% of daytime)
	Written informed consent by the patient.

Exclusion criteria	Known metastatic breast cancer
	Radiotherapy, chemotherapy, immunotherapy, or investigation therapy within the preceding four weeks
	Previous splenectomy or radiotherapy to spleen
	Coexisting or previous other malignancies except *in situ *carcinoma of the cervix or basal cell carcinoma of the skin
	Active uncontrolled infection
	Psychiatric, addictive, or any disorder that compromises ability to give truly informed consent for participation in or to comply with the requirements of the study
	Concurrent systematic corticosteroid treatment
	Autoimmune disease (that is, rheumatoid arthritis, systematic lupus erythematosus; except autoimmune thyroiditis)

**Table 2:** Vaccination (with M-FP or placebo), testing and follow up

	Day	Weeks						Months						Every 3 Months	1 year	Every year
	
	1	2	4	6	8	10	12	4	5	6	7	8	9			
Staging of the disease	+															
Vaccine	+	+	+	+	+	+	+			+			+			
T cell test	+^a^			+^b^			+^c^			+^d^			+^e^		+^f^	+^g, h^
Antibody test	+^a^			+^b^			+^c^			+^d^			+^e^		+^f^	+^g, h^
Clinical examination	+						+			+			+	+		+
Follow-up tests	+									+					+	+
Bone scan and mammography	+															+

For T cell tests and antibody tests sample numbers are highlighted within the box as superscript, indicating time points at which immunological tests were carried out: ^a^first injection; ^b^week 6 (2 weeks after the third injection); ^c^week 12 (2 weeks after the sixth injection); ^d^6 months (3 months after the seventh injection); ^e^9 months (3 months after the eighth) injection; ^f^1 year (3 months after the final [ninth] injection); ^g^2 years (1 year and 3 months after the final injection); ^h^3 years (2 years and 3 months after the final injection). Day 1 is the day of first vaccination. Follow up was every 3 months for 5 years. Antibody test = blood for testing antibody levels; Follow-up tests = laboratory tests, chest X-ray, upper and lower abdominal ultrasound; M-FP, oxidized mannan conjugated to MUC1 fusion protein; T cell test = PBMC for testing IFN-γ; Vaccine = M-FP or placebo administered subcutaneously.

**Table 3:** Follow up of patients injected with M-FP

Patient number	Age (years)	Date of surgery	Type of treatment	Tumour size (cm)	Total nodes	Metastatic nodes	Grade	Date of first injection	Side effects	Recurrence	Last follow up	Follow up (months)
1	58	02/12/97	Partial mast + RT + tam	1.0	17	1	II	21/01/98	Mild skin redness	-	13/06/05	90
2	72	14/01/98	Total mast + tam	2.0	12	1	I	03/02/98	Mild skin redness	-	13/06/05	89
3	52	24/03/98	Partial mast + RT + tam	1.3	20	2	II	16/11/98	-	-	13/06/05	87
4	53	22/05/98	Partial mast + RT + tam	1.5	23	2	III	30/11/98	-	-	15/06/05	85
5	53	03/09/98	Total mast + tam	3.0	21	4	^a^	23/09/98	-	-	15/06/05	81
6	58	10/11/98	Total mast + tam	1.5	22	3	II	30/11/98	Mild skin redness	-	15/06/05	79
7	78	08/12/98	Bilateral total mast +tam	Right 1.2 Left 1.2	18 16	0 4	I I	28/12/98	-	-	17/06/05	78
8	62	27/01/99	Partial mast + RT + tam	1.0	8	1	II	16/02/99	Mild skin redness	-	17/06/05	77
9	61	01/04/99	Partial mast + RT + tam	2.0	18	1	I	26/04/99	-	-	17/06/05	74
10	58	21/04/99	Partial mast + RT + tam	2.0	17	3	II	07/05/99	-	-	20/06/05	74
11	53	13/07/99	Partial mast + RT + tam	2.3	29	1	^a^	19/08/99	Mild skin redness	-	20/06/05	71
12	59	16/09/99	Total mast + tam	1.4	19	1	I	26/06/00	-	-	20/06/05	69
13	59	10/11/99	Total mast + tam	0.7	10	1	^a^	22/11/99	-		22/06/05	67
14	63	09/02/00	Partial mast + RT + tam	1.2	13	1	II	01/03/00	-	-	22/06/05	64
15	55	10/02/00	Partial mast + RT + tam	2.5	23	3	II	25/02/00	-	-	24/06/05	64
16	65	29/06/00	Partial mast + RT + tam	0.8	17	1	II	17/07/00	Mild skin redness	-	24/06/05	60

^a^The histologic type was invasive lobular adenocarcinoma with no grading. mast, mastectomy; M-FP, oxidized mannan conjugated to MUC1 fusion protein; RT, radiotherapy; tam, tamoxifen.

**Table 4:** Follow up of patients injected with placebo

Patient number	Age (years)	Date of surgery	Type of treatment	Tumour size (cm)	Total nodes	Metastatic nodes	Grade	Date of first injection	Side effects	Recurrence	Last follow up	Follow up (months)
17	62	11/12/97	Total mast + tam	2.5	27	1	II	28/01/98	Mild skin redness	-	14/06/05	99
18	54	14/12/97	Partial mast + RT + tam	2.0	20	1	II	10/01/98	-	Local, 14/07/99	14/06/05	99
19	55	16/12/97	Partial mast + RT + tam	1.5	17	1	II	06/05/98	-	-	14/06/05	99
20	60	23/12/97	Partial mast + RT + tam	2.3	24	2	II	13/12/97	-	-	16/06/05	99
21	62	20/01/98	Total mast + tam	2.4	27	2	II	13/03/98	-	-	16/06/05	98
22	61	13/03/98	Total mast + tam	1.8	23	2	I	29/04/98	-	-	17/06/05	97
23	62	01/04/98	Partial mast + RT + tam	1.5	12	1	II	08/07/98	Mild skin redness	-	17/06/05	96
24	54	29/04/98	Partial mast + RT + tam	0.5	17	1	III	22/07/98	Mild skin redness	-	21/06/05	96
25	58	10/11/98	Total mast + tam	2.2	21	3	II	30/01/98	-	Bone, 13/11/00	Death 10/11/03	
26	51	17/02/99	Partial mast + RT + tam	2.0	11	1	II	10/03/99	-	Bone, 28/09/00	21/06/05	86
27	71	10/06/99	Total mast + tam	3.2	26	1	II	01/07/99	-	-	23/06/05	82
28	70	30/06/99	Partial mast + RT + tam	2.0	20	3	II	13/07/99	-	-	23/06/05	82
29	54	02/12/99	Partial mast + RT + tam	2.5	12	1	II	10/01/00	-	-	24/06/05	76
30	81	08/12/99	Total mast + tam	2.5	14	1	III	10/01/00	-	-	27/06/05	75
31	58	24/02/00	Partial mast + RT + tam	2.0	13	3	II	20/03/00	Mild skin redness	Liver, 16/11/00	Death 2002	

mast, mastectomy; M-FP, oxidized mannan conjugated to MUC1 fusion protein; RT, radiotherapy; tam, tamoxifen.
